# Fibrin sealant does not decrease seroma output or time to drain removal following inguino-femoral lymph node dissection in melanoma patients: A randomized controlled trial (NCT00506311)

**DOI:** 10.1186/1477-7819-6-63

**Published:** 2008-06-18

**Authors:** Melinda M Mortenson, Yan Xing, Storm Weaver, Jeffrey E Lee, Jeffrey E Gershenwald, Anthony Lucci, Paul F Mansfield, Merrick I Ross, Janice N Cormier

**Affiliations:** 1 Department of Surgical Oncology, The University of Texas M. D. Anderson Cancer Center, Houston, Texas, USA; 2 Associate Professor of Surgery and Biostatistics, Department of Surgical Oncology-Unit 444, The University of Texas M. D. Anderson Cancer Center, 1515 Holcombe Boulevard, Houston, TX 77030-1402, USA

## Abstract

**Background:**

This study assessed the impact of closed suction drains and evaluated whether the intraoperative use of a fibrin sealant decreased time to drain removal and wound complications in melanoma patients undergoing inguino-femoral lymph node dissection.

**Methods:**

A pilot study (n = 18) assessed the impact of a closed suction drain following inguino-femoral lymph node dissection. A single-institution, prospective trial was then performed in which patients were randomized to a group that received intraoperative application of a fibrin sealant following inguino-femoral lymph node dissection or to a control group that did not receive sealant.

**Results:**

The majority of the patients enrolled felt the drains caused moderate or severe discomfort and difficulties with activities of daily living. Thirty patients were then randomized; the median time to drain removal in the control group (n = 14) was 30 days (range, 13–74) compared to 29 days (range, 11–45) in the fibrin sealant group (n = 16; *P *= 0.6). Major and minor complications were similar in the two groups.

**Conclusion:**

Postoperative closed suction drains were associated with major patient inconvenience. Applying a fibrin sealant at the time of inguino-femoral lymph node dissection in melanoma patients did not reduce the time to drain removal or postoperative morbidity. Alternative strategies are needed.

## Background

Therapeutic inguino-femoral lymph node dissection (ILND) has been associated with clinically significant postoperative morbidities [1-3], including infections, skin flap complications, and lower extremity lymphedema, leading to extended hospitalizations, reduced quality of life, and delayed return to normal activities [4,5]. A 50% incidence of complications following ILND in melanoma patients has been reported, and some studies have indicated that the incidence of short-term (within 30 days of surgery) and long-term morbidity from ILND may be as high as 75% [4-7]. Patients who have comorbidities that compromise their ability to walk, patients who have had complicated incisions for previous operations, obese patients, or patients who have locally advanced disease may experience even higher rates of postoperative morbidity. Despite these risks, therapeutic lymphadenectomy is generally performed for patients with confirmed node-positive stage III melanoma because it is the only potentially curative treatment.

Currently, closed suction drains (CSDs) are inserted at the time of ILND to decrease seroma formation, wound dehiscence, and infection. However, CSDs are not without consequence: they require a high level of maintenance, cause discomfort, interfere with mobility, and serve as potential routes for infection when drainage is prolonged. Therefore, strategies that can be used to prevent postoperative fluid accumulation, thereby reducing the length of time CSDs are in place or even eliminating the use of the drains, potentially will decrease morbidity and increase the quality of life of patients undergoing ILND for melanoma.

One such strategy may be the use of fibrin sealants, which have been used to provide hemostasis as well as tissue apposition and sealing in a wide variety of surgical applications including reconstructive, breast, cardiac, gastrointestinal, and head and neck procedures [8]. While the role of fibrin sealants as hemostatic agents has been well documented [9-11], these substances' ability to reduce fluid accumulation in dissected spaces through tissue adhesion remains unknown. Several mechanisms have been proposed such as the prevention of seromas by sealing open lymphatic channels and adherence of the elevated skin flaps to eliminate surgically created dead spaces [12]. TISSEEL^® ^VH Fibrin Sealant (Baxter Healthcare Corporation, Westlake Village, CA) is a fibrin sealant composed of fibrinogen/factor XIII and thrombin. When the two substances are combined with calcium chloride and applied topically, they produce a fibrin matrix that promotes hemostasis by mimicking the last step of the physiological coagulation cascade [13] and tissue adhesion by stimulating the exudative phase of wound healing [14].

We postulated that applying a fibrin sealant, such as TISSEEL, in patients undergoing ILND for melanoma might decrease the time needed for CSDs, thereby reducing the incidence of postoperative complications and subsequently improving patient comfort. The primary objective of the study was to determine whether the use of a fibrin sealant applied to the operative bed following ILND would result in earlier postoperative drain removal. The secondary objectives were to determine the postoperative morbidity associated with fibrin sealant following ILND and to assess patient valuation of outcomes by performing a cost-benefit analysis using a willingness-to-pay model.

## Methods

Prior to the accrual of patients for the randomized study, a pilot study was performed to assess the patient-reported impact of a CSD following ILND. A survey was given to a group of 18 patients who had undergone ILND at The University of Texas M. D. Anderson Cancer Center within the 6 preceding months. The survey focused on short-term lifestyle changes, aversion behaviors, and expenditures related to having a CSD. Patients were asked to judge the drain's effect on their daily activities on a scale of 1–5, with 1 being "not a problem" and 5 being "a severe problem." The survey also presented patients with several hypothetical scenarios and asked patients to value, in dollars, their willingness-to-pay for potential reductions in the time to drain removal using standard methods [15,16].

### Patients

Following the pilot study, we performed a single-institution, phase III randomized trial. Approval from M. D. Anderson's Institutional Review Board was obtained prior to patient recruitment. Written informed consent was obtained. Patients were eligible for the study if they were scheduled to undergo a superficial ILND with or without a concurrent deep pelvic (iliac/obturator) lymph node dissection or limb perfusion for the treatment of melanoma. Patients were not eligible if they had known hypersensitivity to bovine proteins, had received prior radiation therapy to the operative site, were pregnant or lactating, had been steroid-dependent within the past 6 months, used aspirin or other anti-platelet drugs (excluding celecoxib) within 7 days of the operation, or had pre-existing lymphoma or other pre-existing medical conditions as per the discretion of the principal investigator.

### Technique of lymph node dissection

With the patient positioned in a slight frog-leg position a reverse lazy S incision is made medial to the anterior superior iliac spine extending vertically to the inguinal crease, across the inguinal region, and then vertically down to the apex of the femoral triangle. If a previous biopsy was performed or if palpable disease is present, an ellipse of skin incorporating the previous incision or overlying the palpable disease is included. The borders of flap dissection include the pubic tubercle and the midbody of the adductor longus medially, the lateral edge of the sartorius laterally, and the apex of the femoral triangle inferiorly. All fatty, node bearing tissue is removed and the saphenous vein is divided at the apex of the femoral triangle. Dissection is maintained in the plane of the femoral vessels up to the level of the fossa ovalis. The saphenous vein is suture-ligated at the saphenofemoral junction and the specimen is removed. A frozen-section examination is performed of the lowest iliac node (Cloquet's node). The saphenous vein can be spared in the presence of minimal disease but should be taken in the presence of gross disease in close proximity to or surrounding the vein.

In many cases, especially in obese patients or those with bulky disease, a sartorius muscle transposition is performed by dividing the sartorius muscle at its origin on the anterior superior iliac spine. The muscle is then dissected from its anatomic position preserving the medial vascular bundle and then transposing the muscle over the femoral vessels. Before closure, the skin edges are critically assessed and debrided back to healthy tissue. One or two 19F Blake drains are placed and the incision is closed in a layered fashion with an interrupted dermal layer of absorbable suture and then either staples or an absorbable subcutaneous suture for the skin. Indications for a deep groin dissection (iliac and obturator nodes) include: metastatic disease to Cloquet's node, clinically palpable disease, bulky or suspicious appearing pelvic lymphadenopathy on cross-sectional imaging, or biopsy proven deep inguinal lymph nodes.

### Randomization

Participants were randomized in the operating room using the institutional computerized randomization process. The treatment group received TISSEEL fibrin sealant following ILND, while the control group did not receive fibrin sealant. The results of the randomization were blinded until the surgical dissection was complete.

### Surgical care

After ILND, while patients were still under general anesthesia, TISSEEL (Baxter) was reconstituted according to the manufacturer's instructions and applied locally. Because thrombin concentration determines the setting time of the TISSEEL clot, a diluted thrombin mixture (5 IU/mL) was used to extend this setting time; this was made by diluting the standard preparation of 500 IU/mL with 100 mL of sterile water and 2 mL of 10% calcium chloride. Five milliliters of the diluted thrombin mixture were applied to all of the cavity surfaces. A 19-French Blake drain was inserted through a separate incision site and surgically secured to the skin in all patients. The surgical incision was then closed.

All patients were given the same postoperative instructions, including directions to minimize activity of the involved extremity and permission to shower after 48 hours. The drain was removed once the cumulative serous volume was ≤ 30 mL/24 hours for 2 consecutive days or if 30 days had elapsed since surgery. Patient follow-up was performed at the M. D. Anderson Melanoma and Skin Center or by the patients' primary physicians at 2–4 weeks and again at approximately 6 weeks following surgery. If patients were seen by their primary physicians, clinical information was obtained by the research nurse by telephone.

### Clinical and pathological data

Clinical and pathologic data were collected prospectively beginning at the time of enrollment until 8 weeks postoperatively. These data included age, sex, height, weight, comorbidities, medications, tobacco use, previous treatments for melanoma, indications for ILND, perioperative antibiotics, type of surgical procedure, estimated blood loss, operative time, final pathologic findings, length of time before drain removal, and complications. Major complications were defined as those that required hospital readmission, intravenous antibiotics, or operative debridement. Minor complications included erythema that resolved with oral antibiotics, wound dehiscence that could be managed on an outpatient basis, clinically relevant seromas (requiring subsequent drainage), and drain-related problems that required a visit to the clinic or emergency room. A wound infection was defined as a culture-positive seroma aspiration or clinical evidence of infection (such as fever, erythema, or cellulitis), as judged by a clinician, that required antibiotics or more aggressive wound management.

### Statistical analysis

The primary outcome measure of the study was the time to drain removal. The study size was determined based on institutional experience that without fibrin glue, the median time to drain removal was 21 days. To detect a 7-day decrease in the time to drain removal, we calculated that at least 28 patients needed to be enrolled in the study.

Two-sample comparisons were done using *t-*tests or Wilcoxon rank sum tests for continuous variables and chi square tests for discrete variables [17]. Univariate analyses were performed using linear regression models to identify any factors associated with an increased number of days to drain removal. A log transformation was required to normalize the data (days to drain removal). Computations were carried out with Stata software (Stata/SE version 10 for Windows; StataCorp, College Station, TX). A *P*-value of less than 0.05 was considered statistically significant.

## Results

The 18 pilot study patients' responses to the willingness-to-pay survey are summarized in Table 1. The median length of time before postoperative drains were removed was 20.5 days. Drain care took more than 30 minutes each day for 72% of patients, with an overall median time of 60 minutes. The majority of patients felt the drains caused moderate to severe discomfort (56%) as well as moderate to severe difficulties with daily activities including dressing (67%), bathing (78%), and sleeping (72%). The willingness-to-pay questionnaire showed that patients were willing to pay a median of $175 out-of-pocket for a reduction in drain time of 4 days. Five patients (28%) were willing to pay $500 for a reduction in drain time.

**Table 1:** Summary of Responses from 18 Melanoma Patients Who Had Previous Inguino-femoral Lymph Node Dissection (ILND)

Variable	No.		
Median age, years (range)	56.3 (42–84)		
Sex, male/female, no. patients	9/9		
Median time to postoperative drain removal, days (range)	20.5 (5–35)		
Time of daily care for drain^a^, minutes (range)	60 (10–120)		
No. patients with WTP^a ^= $100	15		
No. patients with WTP^a ^= $500	5		
WTP^a^, dollars (range)	175 (0–550)		

	**No. of patients**	**% of total**	**% of patients ≥ 3**

Discomfort			56
1 = Not a problem	4	22	
2 = Mild problem	4	22	
3 = Moderate problem	7	39	
4 = Moderately severe problem	2	11	
5 = Severe problem	1	6	

Getting dressed			67
1 = Not a problem	3	17	
2 = Mild problem	3	17	
3 = Moderate problem	4	22	
4 = Moderately severe problem	6	33	
5 = Severe problem	2	11	

Bathing			78
1 = Not a problem	3	17	
2 = Mild problem	1	6	
3 = Moderate problem	8	44	
4 = Moderately severe problem	1	6	
5 = Severe problem	5	28	

Sleeping			72
1 = Not a problem	5	28	
2 = Mild problem	0	0	
3 = Moderate problem	7	39	
4 = Moderately severe problem	4	22	
5 = Severe problem	2	11	

Importance of reducing number of days that drain in place			61
1 = Not at all important	4	22	
2 = Slightly important	3	17	
3 = Moderately important	4	22	
4 = Very important	6	33	
5 = Critically important	1	6	

^a^WTP, Willingness-to pay with out-of pocket dollars for a reduction in drain time of 4 days.

Thirty patients were enrolled in the randomized trial between June 17, 2005, and May 30, 2007; 14 patients were randomized to the control group and 16 to the fibrin sealant group. Patient demographics, comorbidities, and preoperative treatment regimens were similar in the two groups (Table 2). More patients in the fibrin sealant group (n = 7) underwent deep pelvic node dissection than in the control group (n = 3), but the difference was not significant. The median length of surgery, estimated blood loss, number of lymph nodes removed, and number of positive lymph nodes did not differ between the two groups (Table 3).

**Table 2:** Demographic and Clinicopathologic Factors in Melanoma Patients Undergoing Inguino-femoral Lymph Node Dissection (ILND)

	**Fibrin sealant group, n = 16**		**Control group, n = 14**		
			
Variable	No.^a^	% of total	No.^a^	% of total	***P *value**
Median age, years (range)	52 (38–91)		60 (18–73)		0.79
Sex, male/female	12/4	75/25	7/7	50/50	0.16
Median BMI, kg/m^2 ^(range)	28.3 (19.6–42.1)		32.3 (21–48.6)		0.15
Comorbidities					
Tobacco use	1	6	2	14	0.58
Diabetes	1	6	4	29	0.16
AJCC tumor stage					1.00
III	14	88	13	93	
IV	2	12	1	7	
Melanoma treatment prior to ILND					
Surgery					0.34
None/FNA	5	31	4	29	
SLN biopsy	6	38	8	57	
Excisional biopsy/WLE	5	31	2	14	
Systemic therapy					0.27
Chemotherapy	2	12	0		0.48
Interferon	1	6	2	14	0.59
Interleukin-2	1	6	0		1.00

BMI, body mass index; AJCC, American Joint Committee on Cancer; FNA, fine needle aspiration; SLN, sentinel lymph node; WLE, wide local excision.

^a^Data are number of patients unless otherwise indicated.

**Table 3:** Surgical Treatments and Outcomes after Inguino-femoral Lymph Node Dissection (ILND)

	**Fibrin sealant group n = 16**		**Control group n = 14**		
			
**Variable**	No.	% of total	No.	% of total	***P *value**
Surgical Treatment					

Patients undergoing superficial ILND	9	56	11	79	0.26
Patients undergoing superficial + deep ILND	7	44	3	21	
Median length of surgery, minutes (range)	227 (95–455)		261 (96–713)		0.53
Median estimated blood loss, mL (range)	50 (0–300)		75 (0–1075)		0.75
Median No. lymph nodes removed (range)	17.5 (9–56)		15 (9–38)		0.07
Median No. positive lymph nodes (range)	3 (1–12)		2 (0–21)		0.41
Patients undergoing concurrent limb perfusion	0	0	2	14	0.21

Outcomes					

Median time to drain removal, days (range)	31 (11–45)		26 (13–74)		0.59
Patients with clinically evident seroma	3	19	3	21	1.00
Patients with clinically evident seroma requiring drainage	3	19	2	14	1.00
Patients with wound infection	7	44	7	50	0.73
Major (inpatient management)	3	19	2	14	1.00
Minor (outpatient management)	4	25	5	36	0.69
Patients with wound dehiscence	7	44	5	36	0.65
Major (inpatient management)	1	6	0	0	1.00
Minor (outpatient management)	6	38	5	36	0.92
Patients with drain issues					
No., number	1	6	3	21	0.32

Postoperatively, there was no statistically significant difference in the time to drain removal between the two groups: the fibrin sealant group required drains for a median of 31 days (range, 11–45 days), compared to 26 days (range, 13–74 days) in the control group (*P *= 0.6).

There was also no significant difference between the groups with respect to overall complications. Three patients in each group had postoperative seromas; except for one seroma in a control-group patient, the seromas required drainage. The incidences of wound infections and wound dehiscence were similar in the two groups. One patient in each group was treated with outpatient wound vacuum placement following wound dehiscence.

Six patients (20%; two in the control group and four in the fibrin sealant group) had major complications requiring hospital readmission, intravenous antibiotics, or operative debridement. Minor complications, including wound infection (36% in the control arm and 25% in the fibrin sealant arm), minor wound dehiscence (36% and 38%), and drain issues (21% and 6%), were also similar in the two groups.

There were three drain complications in the control group: two required visits to the emergency center or outpatient clinic, and one required placement of an additional drain. One patient in the fibrin sealant group had a drain complication that required additional outpatient clinic visits and placement of an additional drain.

Because there were no significant differences between the fibrin sealant group and the control group, the groups were combined for a univariate analysis to evaluate the association between various clinicopathologic and treatment factors and the time to drain removal. There was no association between patient factors (i.e., body mass index or comorbidities), prior systemic therapy, surgical treatment, or postoperative complications and the time to drain removal (Table 4).

**Table 4:** Univariate Analysis of Potential Factors Associated with Increased Time to Drain Removal Following Inguino-femoral Lymph Node Dissection (ILND)^a^

Variable	**Regression Coefficient**	**Standard error**	***P*-value**
Median BMI	0.01	0.01	0.37
Comorbidity			
Tobacco use	-0.03	0.24	0.90
Diabetes	0.04	0.20	0.86
Prior systemic therapy	-0.05	0.18	0.80
Treatment group (fibrin sealant versus control)	3.31	0.11	0.88
Type of surgery (superficial +/- deep pelvic ILND)	-0.01	0.16	0.95
Total no. of lymph nodes removed (< 17 versus ≥ 17)	-0.15	0.14	0.32
No. of positive lymph nodes (< 2 versus ≥ 2)	0.01	0.15	0.95
Wound infection			
Major	-0.02	0.20	0.94
Major plus minor	0.01	0.15	0.95

BMI, body mass index; ILND, inguino-femoral lymph node

## Discussion

The results of the pilot study demonstrated that postoperative CSDs had a major negative impact on patients' daily activities, comfort, and time. In a theoretical scenario, the majority of patients in the pilot study were willing to pay $100–$200 out-of-pocket to reduce the time to drain removal by at least 4 days. Given these findings, a randomized controlled trial was performed using fibrin sealant at the time of ILND to potentially decrease seroma formation and the time to CSD removal. The randomization process was successful with a similar distribution of clinicopathologic factors among the fibrin sealant group and the control group. Our findings, however, do not support the use of a fibrin sealant for this purpose, as it did not affect the time to drain removal for melanoma patients undergoing ILND.

The pathophysiology of seroma formation has not fully been delineated. The majority of literature pertains to breast cancer patients undergoing mastectomy with or without axillary lymph node dissection. In these patients, it has been suggested that a number of anatomical factors play a role in seroma formation including the creation of a large potential dead space, irregularity of the chest wall, and movement of the chest wall secondary to respiration and shoulder movement all of which may prevent flap adherence [18]. Leakage from transected lymphatics is believed to be an important factor in seroma formation although this is supported by only scant evidence. Other studies have suggested that seroma formation may result from an inflammatory exudate [19,20].

Kuroi *et al*., have identified several risk factors for seroma formation in breast cancer patients including obesity, extended radical mastectomy, and large output drain volume in the early postoperative period [21]. Others have found that thermal trauma from electrocautery dissection may increase the incidence of seroma formation, however, alternative techniques such as sharp dissection or ultrasonic scissors may increase operative blood loss [22]. Another surgical technique that may decrease seroma formation is obliteration of the dead space by suture flap fixation although this technique is not widely used [23,24].

Fibrin tissue adhesives, which first became commercially available in Europe in 1978, have been used in numerous surgical procedures [9-11]. To our knowledge, this is the first published prospective trial to assess the benefit of fibrin sealant as a means of decreasing lymphatic drainage in melanoma patients undergoing ILND. The basis for the current study included several studies that used fibrin tissue adhesives in axillary lymph node dissection or modified radical mastectomy for breast cancer [25-30]. Given the technical similarities between these procedures and ILND–all of which include the creation of large skin flaps, transaction of multiple small blood vessels and lymphatics, and an anatomical area at risk for shearing forces–we postulated that the proposed benefits of using fibrin tissue adhesives following axillary surgery would translate to ILND.

Although a number of studies have used fibrin tissue adhesive following breast and axillary surgery, the results have been inconsistent. Several randomized trials have reported a benefit of using fibrin tissue adhesives in axillary lymph node dissection or modified radical mastectomy for breast cancer [26,31-34]. However, a similar number of prospective randomized trials have reported that fibrin sealants are ineffective at preventing seroma formation, decreasing the time to drain removal, or reducing costs in patients undergoing breast procedures [27-30]. Recently, Carless et al. performed a meta-analysis of 11 trials that had used fibrin sealant to prevent seroma formation after breast cancer surgery and found that fibrin sealant did not reduce the rate of postoperative seroma, drainage volume, or length of hospital stay [35]. These findings are similar to ours.

There are several limitations to the current study. First, the study was small, including a total of only 30 patients, which was 80 percent powered to detect a 7 day difference in time-to-drain removal. Second, it is possible that the amount of fibrin sealant applied or the concentration used in this study was not optimal for lymphatic sealing.

Several factors, including safety issues and cost, must be considered when using fibrin sealants. The fibrinogen and thrombin components of TISSEEL are obtained from pooled human plasma from screened donors [8] and thus carries a risk of viral transmission. To avert this risk, a double-vapor heat deactivation procedure is used to eliminate viruses, and polymerase chain reaction testing for viral deoxyribonucleic acid is performed [8,13]. Another safety concern in TISSEEL is that aprotinin, the antifibrinolytic component used to prevent sealant degradation, is from a bovine source, and thus the product is contraindicated in patients who are sensitive to bovine products [36]. Newer, synthetic fibrin sealants have been developed to avoid the risk of allergic reactions. Another promising application of fibrin sealant matrices is the addition of products such as antibiotics or antineoplastics that would allow the fibrin sealant to act as a medium for local delivery of these agents [13,34].

## Conclusion

The application of a fibrin sealant at the time of ILND in patients with melanoma does not significantly influence the time to drain removal, seroma formation, or subsequent postoperative morbidity. Based on the results of this study, the use of fibrin sealant for ILND is not indicated. Alternative strategies that decrease the time to drain removal or eliminate the use of CSDs would be valuable in improving the quality of life of melanoma patients undergoing ILND.

## Abbreviations

CSD: closed suction drains; ILND: inguino-femoral lymph node dissection; SLN: sentinel lymph node; BMI: body mass index

## Competing interests

This study was supported by a research grant from Baxter Pharmaceuticals.

## Authors' contributions

JEL, JEG, PFM, MIR, and JNC contributed to the conception and design of the study, JEL, JEG, PFM, AL, MIR, and JNC contributed to the acquisition of data, MMM and YX contributed to the analysis and interpretation of data, SW participated in the coordination and helped to draft the manuscript. All authors read and approved the final manuscript.
